# Study on the Mechanism of Prunella Vulgaris L on Diabetes Mellitus Complicated with Hypertension Based on Network Pharmacology and Molecular Docking Analyses

**DOI:** 10.1155/2021/9949302

**Published:** 2021-10-15

**Authors:** Xinyi Jiao, Haiying Liu, Qinan Lu, Yu Wang, Yue Zhao, Xuemei Liu, Fang Liu, Yaoyao Zuo, Wenbo Wang, Yujie Li

**Affiliations:** ^1^College of Traditional Chinese Medicine, Shandong University of Traditional Chinese Medicine, Jinan, China; ^2^ChaYeKou Town Health Center of LaiWu District, Jinan, China; ^3^The Second Affiliated Hospital of Shandong University of Traditional Chinese Medicine, Jinan, China

## Abstract

The role of traditional Chinese medicine Prunella vulagaris L in the treatment of tumors and inflammation has been widely confirmed. We found that some signaling pathways of Prunella vulgaris L action can also regulate diabetes and hypertension, so we decided to study the active ingredients, potential targets and signaling pathway of Prunrlla vulgaris L, and explore the “multi-target, multi-pathway” molecular mechanism of Prunella vulgaris L on diabetes mellitus complicated with hypertension(DH). *Methods*. Based on TCMSP(Traditional Chinese Medicine Systems Pharmacology Database and Analysis Platform) and CNKI(China National Knowledge Infrastructure), the components and action targets related to Prunella vulgaris L were screened. The OMIM(Online Mendelian Inheritance in Man) and GeneCards (The human gene database) were used to search for targets related to DH. The “gene - drug - disease” relationship map was drawn by Cytoscape_v3.7.2 plug-in. The target was amplified by the STRING platform, and the “protein - protein” interaction relationship (PPI) network of the interacting target was obtained by the STRING online analysis platform and the Cytoscape_v3.7.2 plug-in. Finally, GO enrichment analysis and KEGG pathway enrichment analysis were conducted on David and Metascape platform to study the co-acting targets. Results. 11 active components, 41 key targets and 16 significant signaling pathways were identified from Prunella vulgaris L. The main active components of Prunella vulgaris L against DH were quercetin and kaumferol, etc, and potential action targets were IL-6 and INS, etc and signaling pathways were AGE-RAGE signaling pathway, TNF signaling pathway, MAPK signaling pathway, PI3K-AKT signaling pathway, etc. It involves in biological processes such as cell proliferation, apoptosis and inflammatory response. *Conclusions*. The main molecular mechanism of Prunella vulgaris L against DH is that sterols and flavonoids play an active role by affecting TNF signaling pathway, AGE-RAGE signaling pathway, MAPK pathway, PI3K-Akt pathway related targets such as IL-6 and INS.

## 1. Introduction

The global prevalence of diabetes mellitus (The following abbreviations are diabetes) has continued to grow in recent years. According to the International Diabetes Alliance, the estimated number of diabetes patients worldwide in 2019 was 463 million, and is increasing year by year [1]. In the pathogenesis of Western medicine, diabetes is a metabolic disease characterized by hyperglycemia due to a deficiency in insulin secretion or insulin dysfunction. Continuous hyperglycemia and long-term metabolic disorders can lead to damage to systemic tissues and organs, especially the eye, kidney, cardiovascular and nervous system, and the cause of which has not been fully clarified. About 70-80% of patients with diabetes eventually die from cardiovascular complications [2], hypertension is one of them, both were associated with insulin resistance.People with diabetes are twice the risk of hypertension as non-diabetic patients, and epidemiological studies prove that hypertension is significantly higher in the diabetic population than in the general population [3],40% ~50% of patients with type 2 diabetes have hypertension and almost 100% when diabetes combined with extensive renal impairment. When diabetes mellitus complicated with hypertension, the incidence and severity of coronary heart disease (including myocardial infarction) increased significantly [4].

In traditional Chinese medicine, diabetes and hypertension are attributed to vertigo, wasting-thirst, deficiency of liver-yin and kidney-yin is the common pathological basis of both [5, 6]. Thirst elimination and dry to hurt the lung and stomach, consume body fluid, Yin blood is damaged, liver-yin and kidney-yin are damaged; liver is easy to fire-evil, damage of pubic fluid, liver-yin and kidney-yin nourish each other, [7]. If the condition can not be effectively controlled, a long disease can cause damage of Yin and Yang, with the loss of Yin and Yang. The onset of the two is not successively divided, can appear at the same time, in the process of the disease development, and affect each other, that is, the current clinical common diabetes mellitus complicated with hypertension (DH).

As science advances, plant drugs are gaining more attention and many natural plant chemicals have shown great clinical prospects for combating complications of diabetes and diabetes. Through the examination of ancient books, it is found that Prunella vulgaris L was first contained in Sheng Nong's herbal classic, the main chemical components include triterpenes, sterols, flavonoids, organic acids, coumarin and other type compounds, whose functions include but not limited to blood pressure lowering, blood sugar lowering, etc. Many researchers have now used Prunella vulgaris L in the clinical treatment of diabetes and hypertension [8–10]. But its specific molecular mechanism has not been clearly clarified.

Network pharmacology is a new method and new theory developed on the basis of network biology and multi-directional pharmacology, and based on the construction and analysis technology of the network. Because diabetes is very easy to cause hypertension, and the high mortality rate after the combination, there are few studies on traditional Chinese medicine in treating diabetes associated with hypertension, so we choose to explore the molecular mechanism of Prunella vulgaris L on DH. For the complex diseases of DH, its multi-target and multi-pathway characteristics are suitable for exploring the molecular mechanism with network pharmacology. This paper aims to study the molecular mechanism and pathway prediction of diabetes complicated with hypertension through network pharmacology, and provide a theoretical basis for the further study of the treatment of DH. Its innovation lies in discussing not only one disease in diabetes or hypertension, but exploring the effect of Prunella vulgaris L on disease treatment when diabetes combines hypertension and its complications, and studying the role of the signaling pathway of Prunella vulgaris L in diabetes and hypertension. The molecular docking technology was used to make the results more accurate. The results can not only explore the effect of the traditional drug Prunella vulgaris L on to DH, but also through the study on other diseases.

## 2. Materials and Methods

### 2.1. Identification, Selection, Search, and Screening of Active Compounds

The TCMSP database (http://tcmspw.com/tcmspsearch.php) includes networks of chemicals, targets and drug targets for 499 Chinese herbal medicines, as well as associated drug target networks, Oral bioavailability (OB) is one of the most important pharmacokinetic parameters in drug absorption, distribution, metabolism, excretion (ADME), indicating the speed and extent to which the active ingredient or active base is absorbed into the body circulation and is absorbed, and the higher the OB value usually indicates the better drug-likeness(DL) of the bioactive molecule of the drug. Due to the poor bioavailability of some traditional Chinese medicine compounds. Therefore, according to the TCMSP database recommended screening indicators combined with pubmed, CNKI and other database reports, OB ≥30% and DL ≥0.18 were identified as screening criteria for active ingredients in Prunella vulgaris L, substances meeting active compounds.

### 2.2. Target Gene Prediction

The Uniprot Protein Database (https://www.uniprot.org/)is the most informative protein sequence database to convert the protein ingredients of Prunella vulgaris L active compounds into gene targets in preparation for the next exploration of the relationship with disease.

### 2.3. Disease Target Gene Finding

The OMIM database (http://www.omim.org/), GeneCards database(http://www.genechttp://ards.org/) is recognized as a disease target database online retrieval tool that can provide a full range of genetic information. In the above database, “diabetic mellitus” “hypertension” was retrieved as the search word, and the intersection and obtained the action targets associated with DH.

### 2.4. Filter the Key Targets

The same fraction of the disease target and Prunella vulgaris L targets were taken using the R language. The results are the key targets for “Prunella vulgaris L-DH”, and draw the veen diagram.

### 2.5. Network Construction

Information on disease targets, drug active ingredient targets and so on was imported into the Cytoscape software (Version3.7.2) to build a “DH-Prunella vuglaris L-compounds-key target” relationship network. The mechanism of Prunella vulgaris L improving DH was explored by the network.

### 2.6. Build the PPI Network

The key targets of “drug-disease” were imported into the STRING data online analysis platform (https://string-db.org) to expand the gene. The STRING data online analysis platform is a tool capable of analyzing protein interaction networks, able to visualize the relationship between proteins to obtain PPI network maps where high connectivity is called central genes.

### 2.7. Gene Ontology (GO) Term and Kyoto Encyclopedia of Genes and Genomes (KEGG) Pathway Enrichment Analyses

The GO database classifies genes and gene products in three aspects: biological processes, molecular function, and cell components. The KEGG database is a database integrating information about genomic, chemical, and system functions that presents the signaling pathways in which the screened key genes are located. GO and KEGG analysis of the screened key targets using David (https://david.ncifcrf.gov/), Metascape (http://metascape.org/).

### 2.8. Molecular Docking

Download the target protein structure from the PDB database (http://www.rcsb.org/) and the 2D structure of molecular ligands can be downloaded from the PubChem database (https://pubchem.ncbi.nlm.nih.gov/), preprocessing and molecular docking of the target protein and active ingredients using Maestro11.5.

## 3. Results

### 3.1. Inquiry and Screening of Active Compounds

Search TCMSP database for “Prunella vulgaris L”, set the limits of OB ≥30%, OL ≥0.18 and obtain 11 eligible active compounds such as quercetin and Vulgaxanthin-I (Table 1). It can be considered that the oral bioavailability and drug-likeness is good for these 11 compounds. Myrcene, lauric acid, caffeic acid, phellandrene etc. were abandoned due to inadequate filter criteria.

### 3.2. Obtaining Intersection Targets

**Figure 1:**
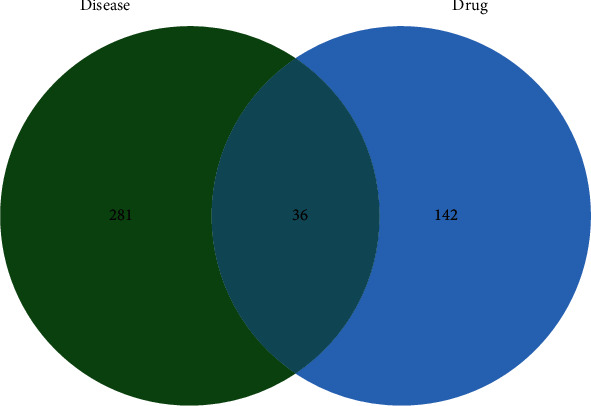
Interintersection of diabetes and hypertension overlap with Prunella vulgaris L.

**Figure 2:**
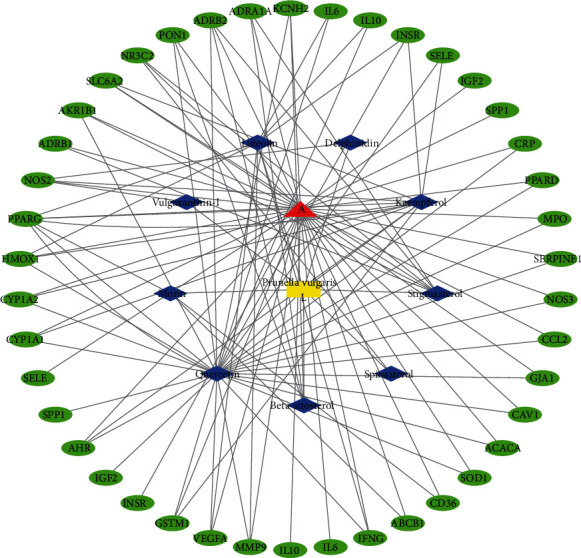
“disease-target-drug- compounds” network diagram of Prunella vulgaris L in the treatment of DH.

The 162 gene targets for the drug active ingredient were obtained through the UNIPROT database gene pairing.The disease targets were crossed between the GeneCards database and 281 gene targets were obtained from the OMIM database. Use R language to map the “disease-drug target” intersection and obtain VENN map (Figure 1), and obtained 36 intersection targets for drugs and diseases. The 36 key targets were imported into the Cytoscape to visualize the “disease-target-drug-compounds” relationship network (Figure 2). In the figure, the red node A represents the DH, the yellow node represents the Prunella vulgaris L, the blue node is the drug active compounds, and the green node is the key target of the disease and drug active compounds.

**Figure 3:**
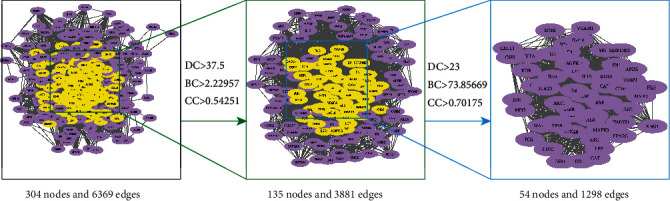
Topology analysis process.

**Figure 4:**
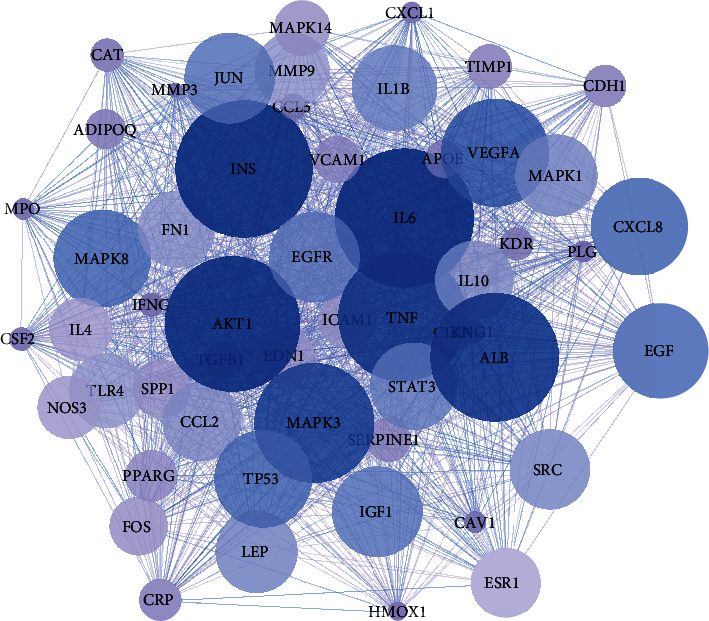
The PPI network diagram.

The 36 key targets were introduced into the STRING analysis platform to obtain 312 genes, hide the disconnected nodes in the network, import the Cytoscape to get a diagram of 304 points, 6369 lines, Use the Cytoscape tool for topological analysis to obtain relevant parameters, selection Degree-centrality(DC), and Betweenness-Centrality(BC), Closeness-Centrality(CC). Set 135 points of DC>37.5, CC>2.5251,3881 lines, BC>73.85669, CC>0.7070175, select 41 core genes and obtain PPI network diagram of 41 points, 748 lines (Figure 3). PPI network diagram of 41 gene targets via Cytoscape (Figure 4).

### 3.3. GO Enrichment Analysis

**Figure 5:**
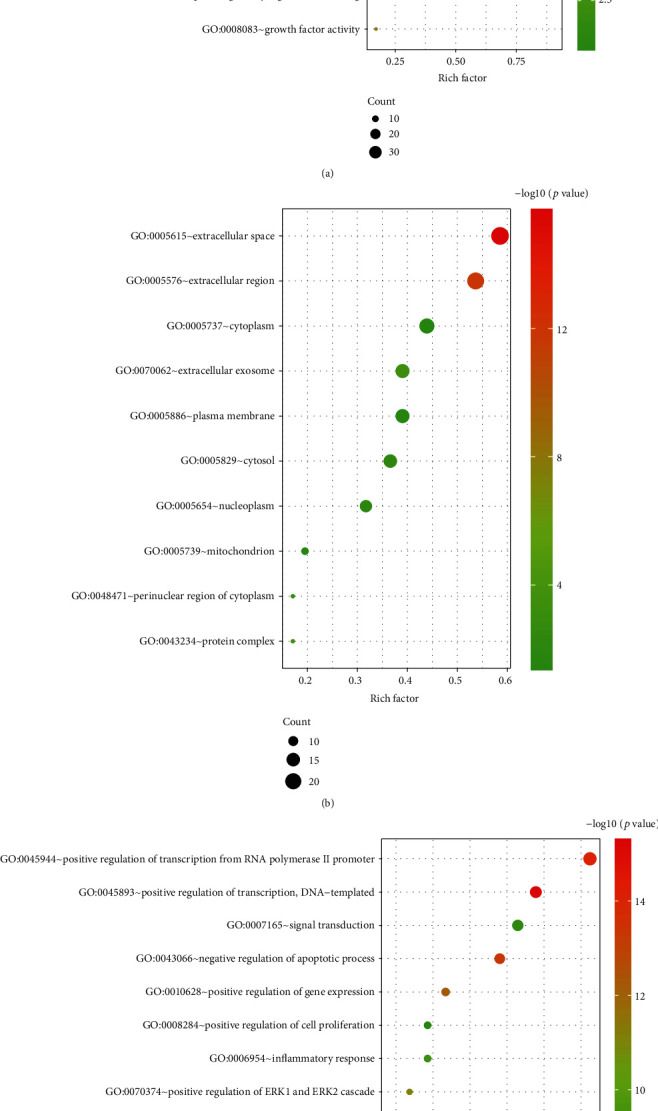
GO enrichment analysis bubble diagram: (a) molecular function; (b) cellular component; (c) biological process.

GO enrichment analysis of 41 target genes through the David database. Making three partial bubble diagram of biological process and cellular component, molecular function at the top 10 enrichment, respectively (Figure 5). It can be seen that the biological process is mainly related to cell proliferation, apoptosis and inflammatory response.

### 3.4. KEGG Analysis

**Figure 6:**
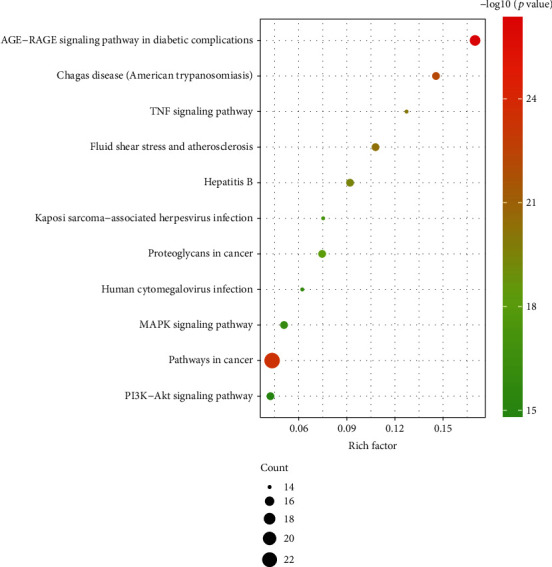
KEGG enrichment analysis.

KEGG enrichment information was obtained through the enrichr database (https://maayanlab.cloud/Enrichr/) and the top 11-bit bubble diagram (Figure 6) shows that the signaling path mainly includes TNF, AGE-RAGE, MAPK, and PI3-Akt.

### 3.5. Molecular Docking Verification

**Figure 7:**
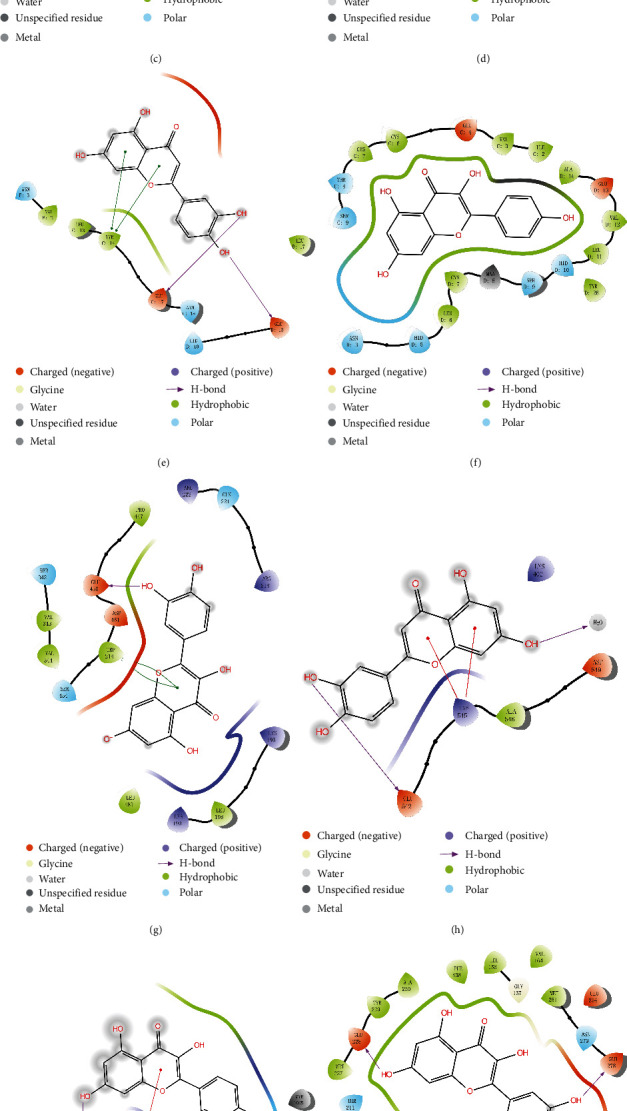
2 D structure of 12 docking results: (a) IL6 with quercetin; (b) IL6 with luteolin; (c) IL6 with kaempferol; (d) INS with quercetin; (e) INS with luteolin; (f) INS with kaempferol; (g) ALB with quercetin; (h) ALB with luteolin; (i) ALB with kaempferol; (j) AKT1 with quercetin; (k) AKT1 with luteolin; (l) AKT1 with kaempferol.

Molecular docking of key components using the software Maestro11.5, Verify the combination of IL6, INS, ALB, AKT1 and quercetin, luteolin, kaempferol separately (Figure 7), calculate the combined energy. The validation is as shown in Table 2, compounds and target proteins are rated as kcal/mol. The lower the binding energy, the better the stability. It is shown that these components can bind to the active site of the protein target.

## 4. Discussion

Diabetes has now become a killer of human health, and the mortality rate of diabetes associated with hypertension is far higher than that of simple diabetes.In Chinese medicine, diabetes and hypertension belong to the category of vertigo and wasting-thirst, and liver-yin and kidney-yin deficiency is their common pathogenesis, so the same traditional Chinese medicine can be used for treatment. Prunella vulgaris L from the “Sheng Nong's herbal classic”, its pharmacological effects include blood pressure, blood sugar, antiviral and other effects. Currently research is the blood pressure-lowering effect of Prunella vulgaris L. For its hypoglycemic effect is often ignored.This paper analyzes the effective components of Prunella vulgaris L, and creates new ideas for the treatment of DH.

This paper analyzes the effective components of Prunella vulgaris L, and creates new ideas for the treatment of DH. The results of this study show that the key compounds that Prunella vulgaris L plays are quercetin, morin, luteolin, kaempferol, beta-sitosterol, delphinidin, spinasterol, vulgaxanthin-I, etc. Studies show that quercetin is a flavonoid,Choi,S et al. confirmed that quercletin can acute enhance acetylcholine-induced 2K1C hypertensive rat vascular relaxation, suggesting that quercletin plays an anti-hypertensive role by reducing vascular elasticity [11]. Meanwhile, it can inhibit the activity of disaccharidase to achieve the hypoglycemic effect [12]. It is also shown that quercetin can stimulate insulin release and inhibit INS-1beta cell activity, and long-term applications can inhibit cell proliferation and induce apoptosis, most likely achieved by inhibiting PI3K/Akt signaling [13]. Quercetin has been proposed to restore the functional quality of pancreatic *β* cells through multiple targeting, and therefore can be attributed to a prospective treatment strategy for diabetes [14]. WuYT et al. found that luteolin reduces blood pressure-lowering vascular smooth muscle cell proliferation and migration through the growth factor-*β* receptor 1 (TGFBR1) pathway, and has anti-oxidation and anti-inflammatory activity, improving glucose metabolism by enhancing insulin sensitivity and improving *β* cell function and quality[15, 16]. Extensive studies have shown that plant sterols such as *β*-glusterol and Spinasterol can prevent and treat hypertension [17, 18]. The researchers found that delphinidin's anti-diabetes mechanism with hypertension may be associated with its strong antioxidant activity, inhibiting *α*-glucossidase and *α*-amylase, angiotensin converting ase (ACE) and direct vascular relaxation or calcium channel regulation [19]. Lai Dengni et al. found that pancreatic *β* cells were immune from apoptosis caused by high glucose stress via the AMPK signaling pathway [20].

We found that the top two gene target, IL6, is a multifunctional cytokine secreted by monocyte macrophages, functioning mainly in immunomodulation. According to research, diabetes, hypertension and other diseases can lead to higher IL6 levels in serum, while multiple reports say that genetic polymorphisms of IL6 are closely related to insulin levels and large blood vessels, and may regulate insulin secretion through pancreatic *α* cells [21]. IL6 also acts as an inflammatory factor whose expression promotes cardiac fibroblasts, thereby increases collagen synthesis and eventually leading to myocardial fibrosis [22]. INS is a candidate susceptibility gene for diabetes [23]. Carmody D et al. argue that mutations in the INS gene can cause hyperglycemia, hyperinsulinemia, etc. Its dominant mutations can produce a translation product causing an unfolded protein response, causing the endoplasmic reticulum stress and eventually apoptosis and diabetes [24]. Many of the metabolic and angiokinesis actions of INS are mediated by activation of phosphatidylinositol 3-kinase (PI3K) and downstream signaling pathways [25]. The researchers found that INS affected vascular tension by its metabolic effects on endothelial cells [26].

In the AGE/RAGE signaling pathway, AGE and its receptor RAGE interactions trigger oxidative stress, inflammatory response, and thrombosis, thus participating in vascular aging and damage, and may be an important cause of diabetes associated with hypertension [27]. AGE/RAGE signaling induces the activation of multiple intracellular signaling pathways involving NADPH oxidase, protein kinases C and MAPK, enhancing NF-*κ* Bactivity to promote a variety of genes associated with atherosclerosis such as IL-6 [28]. The results of these signaling transduction are possible mechanisms for triggering complications of diabetes. For example, it is reported that AGEs can up-gulate the expression of RAGE by activating NF-*κ*B [29]. As previously described, activated NF-*κ*B binds to a specific DNA sequence, regulates transcription of corresponding genes and accelerates the occurrence of cardiovascular complications. It is conceivable that positive feedback cycles between downstream paths may produce a vicious cycle that promotes cardiovascular complications of diabetes.

TNFR1 in the TNF signaling pathway mainly regulates cell regulation processes and is associated with insulin resistance and the pathogenesis of type 2 diabetes [30].The study showed that TNF-*α* levels increased significantly at the generation of oxidative stress [31]. TNF-*α*, as one of the regulators, induces the production of cytokines like IL-1*β*, IL-6 involved in oxidative stress and inflammatory response processes.

The Insulin pathway activates the MAPK pathway that causes vasoconstriction through endothelial-dependent mechanisms [32]. A large number of studies show that MAPK signaling pathway participates in biological cell cycle regulation, cell differentiation, metabolism and other processes. In an important process of hypertension, the biological property changes of vascular smooth muscle cells and vascular endothelial progenitor cells are all regulated by MAPK [33]. The MAPK pathway and the PIK3-AKT pathway can collaborate to regulate TNF-*α* expression and apoptosis [34].

## 5. Conclusion

This paper analyzes the treatment of Prunella vulgaris L for DH, and verifies the docking activity of small molecular ligand and protein with molecular docking. The eleven active components of Prunella vulgaris L for diabetes combined with hypertension include steroids, flavonoids, etc., 41 key targets IL-6, NOS3, etc., and 21 significant signaling pathways, such as AGE-RAGE, HIF-1, etc., and analyzed the specific role of these targets and signaling pathways. It theoretically explains the potential mechanism of traditional Chinese medicine Prunella vulgaris L in treating diabetes with hypertension, and provides the direction for the subsequent effect of Prunella vulgaris L in the treatment of DH.

## Figures and Tables

**Table 1:** Active ingredients of Prunella vulgaris L.

Mol ID	Molecule name	Molecular weight	OB (%)	DL
MOL000358	beta-sitosterol	414.79	36.91	0.75
MOL000422	Kaempferol	286.25	41.88	0.24
MOL004355	Spinasterol	412.77	42.98	0.76
MOL000449	Stigmasterol	412.77	43.83	0.76
MOL004798	Delphinidin	303.26	40.63	0.28
MOL000006	Luteolin	286.25	36.16	0.25
MOL006767	Vulgaxanthin-I	339.34	56.14	0.26
MOL006772	Poriferasterol monoglucoside_qt	412.77	43.83	0.76
MOL006774	Stigmast-7-enol	414.79	37.42	0.75
MOL000737	Morin	302.25	46.23	0.27
MOL000098	Quercetin	302.25	46.43	0.28

**Table 2:** Docking score.

Key ingredients	Docking score		
	Quercetin	Luteolin	Kaempferol
IL6	-6.955	-6.225	-6.102
INS	-5.169	-3.724	-4.907
ALB	-5.022	-4.964	-4.505
AKT1	-6.411	-3.607	-5.940

## Data Availability

The data used to support the findings of this study are available from the corresponding author upon request.

## References

[1] Saeedi P., Petersohn I., Salpea P. (2019). Global and regional diabetes prevalence estimates for 2019 And projections for 2030 and 2045: Results from the International Diabetes Federation Diabetes Atlas. *Diab Res Clin Pract*.

[2] Umamahesh K., Vigneswari A., Surya Thejaswi G., Satyavani K., Viswanathan V. (2014). Incidence of cardiovascular diseases and associated risk factors among subjects with type 2 diabetes - An 11-year follow up study. *Indian Heart, Journal*.

[3] Petrie J. R., Guzik T. J., Touyz R. M. (2018). Diabetes, hypertension, and cardiovascular disease:clinical insights and vascular mechanisms. *CanCardiol.*.

[4] Yang X. (2011). Pathogenesis, etiology and pathogenesis of diabetic hypertension. *Chinese Community Physicians*.

[5] Wang J., Xiong X., Liu W. (2014). Traditional chinese medicine syndromes for essential hypertension: a literature analysis of 13,272 patients. *Evidence-based Complementary and Alternative Medicine*.

[6] Yang H. (2021). *Treatment of diabetes is the combination of spleen, liver and kidney*.

[7] Wendong S., Yutong Z. (2021). Analysis of Wu Jutong yin protection for diabetes. *Beijing Traditional Chinese Medicine*.

[8] Hwang S. M., Lee Y. J., Yoon J. J. (2012). Prunella vulgaris L suppresses HG-induced vascular inflammation via Nrf2/HO-1/eNOS activation. *International Journal of Molecular Sciences*.

[9] Li H. M., Kim J. K., Jang J. M., Kwon S. O., Cui C. B., Lim S. S. (2012). The inhibitory effect of Prunella vulgaris L L. on aldose reductase and protein glycation. *J Biomed Biotechnol*.

[10] Yunfeng C. (2021). Clinical effect analysis of Prunella vulgaris Lsoup on hypertension in the elderly. *Chinese Community Physician*.

[11] Choi S., Ryu K. H., Park S. H. (2016). Direct vascular actions of quercetin in aorta from renal hypertensive rats. *Kidney Res. Clin. Pract.*.

[12] Eid H. M., Haddad P. S. (2017). The antidiabetic potential of quercetin: underlying mechanisms. *Current Medicinal Chemistry*.

[13] Kittl M., Beyreis M., Tumurkhuu M. (2016). Quercetin stimulates insulin secretion and reduces the viability of rat INS-1 Beta-cells. *Cellular Physiology and Biochemistry*.

[14] Oh Y. S. (2015). Plant-derived compounds targeting pancreatic beta cells for the treatment of diabetes. *Evid. Based Complement. Altern. Med.*.

[15] Wu Y. T., Chen L., Tan Z. B. (2018). Luteolin inhibits vascular smooth muscle cell proliferation and Migration by inhibiting TGFBR1 signaling. *Frontiers in Pharmacology*.

[16] Daily J. W., Kang S., Park S. (2021). Protection against Alzheimer's disease by lu teolin: role of brain glucose regulation, anti-inflammatory activity, and the gutmicrobiota liver-brain axis. *BioFactors*.

[17] Yuankun C., Ao Z., Luo Z. (2021). Progress in the pharmacological action of *β*-glusterol. *Journal of Guangdong Pharmaceutical University*.

[18] Babu S., Krishnan M., Rajagopal P. (2020). Beta-sitosterol attenuates insulin resistance in adipose tissue via IRS-1/Akt mediated insulin signaling in high fat diet and sucrose induced type-2 diabetic rats. *European Journal of Pharmacology*.

[19] Da-Costa-Rocha I., Bonnlaender B., Sievers H., Pischel I., Heinrich M. (2014). Hibiscus sabdariffa L. - A phytochemical and pharmacological review. *Food Chemistry*.

[20] Dengni L., Mingyong H., Lingyan Z. (2019). Delphinidin-induced autophagy protects pancreatic *β* cells against apoptosis resulting from high-glucose stress via AMPK signaling pathway. *Acta Biochimica et Biophysica Sinica*.

[21] Ellingsgaard H., Hauselmann I., Schuler B. (2011). Interleukin-6 enhances insulin secretion by increasing glucagon-like peptide-1 secretion from L cells and alpha cells. *Nature Medicine*.

[22] Wang A. W., Song L., Miao J. (2015). Baicalein attenuates angiotensin II-induced cardiac remodeling via inhibition of AKT/mTOR, ERK1/2, NF-*κ*B, and calcineurin signaling pathways in mice. *American Journal of Hypertension*.

[23] Pugliese A., Miceli D. (2002). The insulin gene in diabetes. *Diabetes/Metabolism Research and Reviews*.

[24] Carmody D., Park S. Y., Ye H. (2015). Continued lessons from the INS gene: an intronic mutation causing diabetes through a novel mechanism. *Journal of Medical Genetics*.

[25] Sowers J. R. (2004). Insulin resistance and hypertension. *American Journal of Physiology. Heart and Circulatory Physiology*.

[26] Zeng G., Quon M. J. (1996). Insulin-stimulated production of nitric oxide is inhibited by wortmannin. Direct measurement in vascular endothelial cells. *J Clin Invest*.

[27] Yamagishi S. (2011). Role of advanced glycation end products (AGEs) and receptor for AGEs (RAGE) in vascular damage in diabetes. *Experimental Gerontology*.

[28] Fei J., Ling Y. M., Zeng M. J., Zhang K. W. (2019). Shixiang plaster, a traditional Chinese medicine, promotes healing in a rat model of diabetic ulcer through the receptor for advanced glycation end products (RAGE)/nuclear factor kappa B (NF-*κ*B) and vascular endothelial growth factor (VEGF)/vascular cell adhesion Molecule-1 (VCAM-1)/endothelial nitric oxide synthase (eNOS) signaling pathways. *Medical Science Monitor*.

[29] Zhu W., Li W., Silverstein R. L. (2012). Advanced glycation end products induce a prothrombotic phenotype in mice via interaction with platelet CD36. *Blood*.

[30] Wu S., Dong K., Wang J., Bi Y. (2018). Tumor necrosis factor alpha improves glucose homeostasis in diabetic mice independent with tumor necrosis factor receptor 1 and tumor necrosis factor receptor 2. *Endocrine Journal*.

[31] Verma M. K., Jaiswal A., Sharma P., Kumar P., Singh A. N. (2019). Oxidative stress and biomarker of TNF-*α*, MDA and FRAP in hypertension. *Journal of Medicine and Life*.

[32] Araujo J. E. D. S., Miguel-Dos-Santos R., Macedo F. N. (2020). Effects of high doses of glucocorticoids on insulin-mediated vasodilation in the mesenteric artery of rats. *PLoS One*.

[33] Chen H. X., Xu X. X., Tan B. Z., Zhang Z., Zhou X. D. (2017). MicroRNA-29b inhibits angiogenesis by targeting VEGFA through the MAPK/ERK and PI3K/Akt signaling pathways in endometrial carcinoma. *Cellular Physiology and Biochemistry*.

[34] Li M., Ye J., Zhao G. (2019). Gas6 attenuates lipopolysaccharide-induced TNF-*α* expression and apoptosis in H9C2 cells through NF-*κ*B and MAPK inhibition via the Axl/PI3K/Akt pathway. *International Journal of Molecular Medicine*.

